# Time‐Resolved Native Mass Spectrometry Reveals Reversible Light‐Driven Oligomerization of *Arabidopsis* Cryptochrome 1 and Its Antagonism by BIC1

**DOI:** 10.1002/anie.202525792

**Published:** 2026-04-07

**Authors:** Alicia Just, Nils Niemann, Petra Gnau, Dennis Kock, Thomas Heimerl, Stephan Kiontke, Lars‐Oliver Essen, Alfred Batschauer, Nina Morgner

**Affiliations:** ^1^ Institute of Physical and Theoretical Chemistry Johann Wolfgang Goethe University Frankfurt Germany; ^2^ Department of Biology Philipps University Marburg Germany; ^3^ Department of Chemistry Philipps University Marburg Germany; ^4^ SYNMIKRO Research Center and Department of Chemistry Philipps University Marburg Germany

**Keywords:** blue light inhibitors of cryptochromes, cryptochromes, native mass spectrometry, oligomerization dynamics, photoreceptors

## Abstract

Cryptochromes (CRYs) are blue‐light photoreceptors that mediate light‐dependent signaling in plants. Here, we uncover the molecular mechanism underlying blue‐light activation of the *Arabidopsis thaliana* cryptochrome 1 photolyase homology region (CRY1‐PHR) using time‐resolved native mass spectrometry combined with kinetic modeling. This approach enables direct monitoring of light‐driven complex formation with temporal and molecular resolution. We show that blue‐light activation of CRY1‐PHR follows a reversible assembly pathway in which monomers rapidly form dimers that further assemble into tetramers. A quantitative two‐step kinetic model captures the dynamic interplay between light‐induced oligomerization and thermal disassembly. Strikingly, ATP accelerates tetramer formation and stabilizes oligomers by tuning the underlying photochemistry of the flavin adenine dinucleotide (FAD) chromophore. In contrast, the Blue‐light Inhibitor of Cryptochromes 1 (BIC1) acts as a potent antagonist. BIC1 binds to CRY1‐PHR even in the dark, with significantly increased affinity under blue light, thereby inhibiting oligomerization and actively disassembling pre‐formed tetramers. This disassembly is light‐independent and occurs regardless of CRY's redox state. Together, these findings provide a kinetic and mechanistic framework for reversible blue‐light signaling by plant CRYs and highlight how opposing regulators precisely modulate photoreceptor activation at the molecular level.

## Graphical abstract

Time‐resolved native mass spectrometry uncovers the blue‐light‐dependent kinetic mechanism of *Arabidopsis* CRY1 oligomerization, proceeding sequentially from monomers to dimers and tetramers. ATP promotes assembly, whereas BIC1 actively blocks and disassembles tetramers independent of light, providing mechanistic insight into the regulation of cryptochrome signaling.

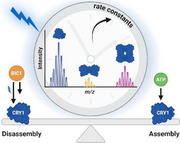

## 1 Introduction

Plants rely on light not only as an energy source but also as a critical environmental signal that guides nearly every aspect of their growth and development [1, 2, 3]. To perceive and translate light information across the spectrum, plants have evolved a sophisticated network of photoreceptors, including phytochromes, phototropins, the UV‐B photoreceptor UVR8, and cryptochromes (CRYs) [1, 4, 5, 6]. Among these, CRYs are evolutionarily conserved flavoproteins that absorb blue and ultraviolet‐A light via a flavin adenine dinucleotide (FAD) chromophore, which is bound non‐covalently within the photolyase homology region (PHR) domain [7, 8, 9, 10]. The PHR domain is complemented by the cryptochrome C‐terminal extension (CCE) that is much less conserved and varies in length and sequence among CRYs [11]. In *Arabidopsis thaliana*, two major CRYs, CRY1, and CRY2, operate in partially overlapping but distinct physiological contexts [12]. CRY1 predominantly controls the early seedling stage, where it suppresses hypocotyl elongation, whereas CRY2 regulates flowering time by acting as a photoperiod sensor [12, 13, 14, 15, 16]. Activation of plant CRYs is initiated by light‐driven photoreduction of FAD [17, 18], which induces conformational changes that promote oligomerization [19, 20, 21, 22] and binding to downstream signaling partners [23]. Although CRY1 and CRY2 are structurally related [24], their functional specialization suggests that they may differ in their activation dynamics, signaling interactions, and mechanisms of regulation. Structural studies have demonstrated that both CRY1 and CRY2 undergo blue light‐induced oligomerization [13, 15, 16, 17], and in the case of CRY2, tetrameric assemblies have been linked to signaling activity [25]. CRY1 likewise forms light‐induced oligomers, as revealed by structural analyses [26], yet the dynamics and regulation of this process are poorly understood. While oligomerization is mediated by the PHR domain, the CCEs are engaged in liquid–liquid phase separation (LLPS) [11]. To distinguish specific oligomerization from the formation of dynamic size‐variable condensates, in vitro studies of the isolated PHR domain are essential to uncover the intrinsic mechanism of CRY oligomerization without the interfering influence of LLPS. In particular, the molecular mechanism underlying CRY1 oligomerization, as well as the influence of cellular cofactors or inhibitory proteins on this process, remains poorly understood.

A major class of negative regulators of CRYs is the Blue‐light Inhibitors of Cryptochromes (BICs) [27]. BICs interact with CRY2 in its PHR domain and efficiently block tetramerization, thereby suppressing signaling [21, 24]. CRY1 has also been identified as a target of BIC1 [21, 25, 26, 27], but the mechanistic details of this inhibition are not resolved. Previous studies demonstrated that BIC1 may associate with CRY1 in both dark and light conditions [28], raising the question whether BIC1 primarily prevents oligomer formation or whether it can actively dismantle pre‐assembled CRY1 complexes.

Another layer of potential regulation arises from cellular metabolites [29, 30, 31]. ATP is known to bind the PHR domain of plant CRYs [17, 18], and a variety of studies have reported that ATP accelerates FAD photoreduction and stabilizes reduced states [30, 32, 33, 34]. However, whether ATP directly influences CRY1 oligomerization dynamics has remained unclear.

Building on our previous work, where we demonstrated time‐resolved investigation of light‐induced conformational changes in the monomeric animal‐like CRY from *Chlamydomonas reinhardtii* (*Cr*aCRY) [35], we now take one step further and explore the oligomerization process that follows photoactivation in CRY1 from *A. thaliana*. We combined different native mass spectrometry techniques, absorption spectroscopy, and negative‐stain transmission electron microscopy (TEM), to unravel the light‐induced assembly and regulation of *Arabidopsis* CRY1 PHR domain in vitro. We show that CRY1 oligomerization proceeds via a sequential monomer → dimer → tetramer pathway that can be quantitatively described by a reversible two‐step kinetic model. ATP accelerates this process, boosting the oligomerization rates and stabilizing the oligomeric states. Furthermore, we demonstrate that BIC1 binds to CRY1 both in darkness and under blue light illumination, with significantly different affinities. Importantly, BIC1 not only prevents tetramer assembly but also disassembles preformed CRY1 oligomers, a property that extends to the constitutively tetrameric CRY1^L407F^ mutant [36, 37]. Finally, we show that BIC1 inhibition does not affect the photoreduction of the FAD chromophore, indicating that BIC1 regulates CRY1 through direct competition with CRY1‐CRY1 contacts rather than by substantially affecting the photochemistry of the chromophore.

Together, our findings establish a quantitative kinetic framework for plant CRY1 oligomerization and reveal dual regulatory inputs: ATP as a bound cofactor and active disassembly by BIC1. These results uncover fundamental principles of CRY1 regulation that distinguish it from CRY2 and highlight new mechanisms by which plant cells integrate cofactor binding and the effect of inhibitory proteins to fine‐tune CRY signaling.

## 2 Results and Discussion

### 2.1 Oligomerization Mechanism of *Arabidopsis* CRY1

**FIGURE 1:**
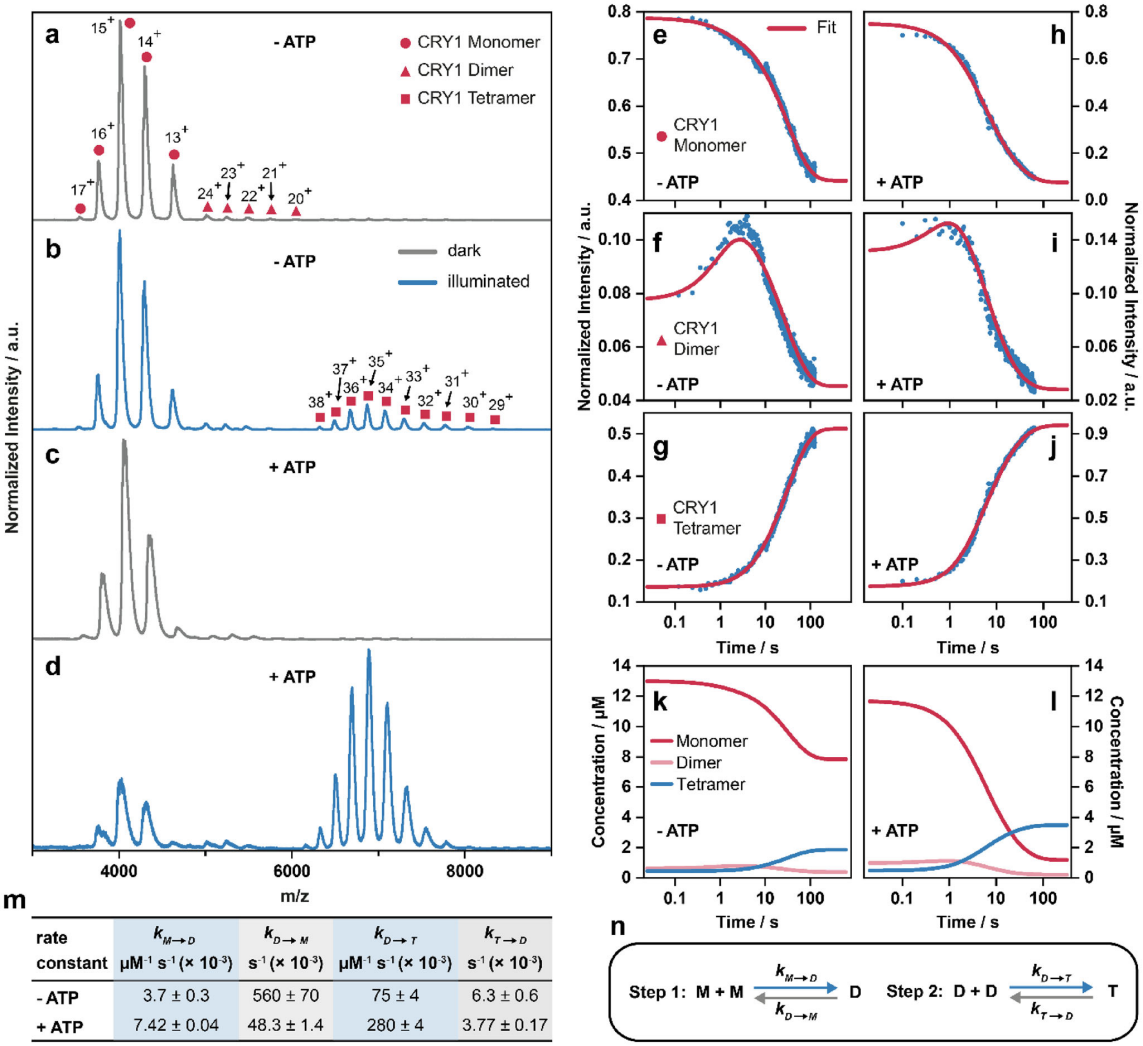
Oligomerization kinetics of CRY1 under blue light and the influence of ATP. nESI‐MS spectrum of CRY1 in the dark (grey line) (a, c) and under illumination with blue light (blue line) (b, d) in absence (a, b) and presence of 100 µM ATP (c, d). Exposure to blue light leads to oligomerization of CRY1, and the addition of ATP promotes this process. Time‐resolved mass spectrometry measurements of the CRY1 monomer (e), dimer (f), and tetramer (g) in absence and presence (h–j) of ATP and the fit (pink line). The time axis is displayed on a logarithmic scale to better resolve the early kinetic transitions. Concentration profile of CRY1 monomer, dimer, and tetramer in absence (k) and presence (l) of 100 µM ATP during exposure to blue light obtained from the fit of the two‐step reversible kinetic model (n). (m) Association (highlighted in blue) and dissociation (highlighted in grey) rates obtained from kinetic modeling in presence and absence of 100 µM ATP. The rates represent the mean of three independent measurements (*n* = 3); error estimates are derived from fitting the kinetic model.

To gain mechanistic insight into *Arabidopsis* CRY1 oligomerization, we investigated the oligomeric states of the recombinant CRY1 PHR domain (CRY1‐PHR), hereafter named CRY1, in vitro by nano electrospray ionization mass spectrometry (nESI‐MS). For these experiments, the nESI setup was equipped with a blue‐light LED, positioned to illuminate the sample directly during the measurement (Figure S1a). Consistent with previous reports [26] CRY1 exists predominantly as a monomer in the dark (*m*/*z*  =  3500–4800, Figure 1a) with only a small fraction present as dimers (*m*/*z*  =  5000–5700). Exposure to blue light promotes its oligomerization into a tetrameric form (*m*/*z*  =  6000–8500) (Figure 1b).

To elucidate the underlying mechanism of this light‐induced oligomerization, we next examined whether tetramer assembly occurs exclusively via dimer intermediates or involves trimeric species as transitional state. To address this, we performed time‐resolved mass spectrometry at room temperature, allowing us to detect and quantify transient oligomeric species under blue light conditions (Figure S2). The time‐resolved data revealed a well‐defined kinetic progression of CRY1 oligomerization (Figures 1e–g and S3a–c). In the initial dark‐adapted state, the CRY was predominantly monomeric (Figures 1a,e and S3a). Upon exposure to blue light, we observed a rapid decrease in monomer abundance (Figures 1e and S3a), accompanied by the appearance of dimers (Figures 1f and S3b). Tetramer formation was observed after a short delay, consistent with a stepwise assembly pathway (Figures 1g and S3c). Dimer levels peaked after approximately 3 s (Figures 1f and S3b), while tetramer accumulation progressed more slowly and reached a stationary plateau after 120 s (Figures 1g and S3c). Notably, the monomeric species did not fully disappear (Figures 1b,e and S3a), suggesting that oligomerization is opposed by a concurrent thermal dissociation process. This observation shows that even under sustained light activation, reversible oligomer assembly and disassembly are in kinetic competition.

To quantitatively describe the oligomerization mechanism, we propose a reversible kinetic model comprising two steps, as shown in Figure 1n. This kinetic model assumes sequential assembly, from monomer via dimer to tetramer (M→D→T), with no direct conversion from monomer to tetramer and no trimeric species. Fitting our data with this model (Figures 1e–g and S3a–c) yielded association and dissociation rate constants listed in Figure 1m. The model accurately described the experimental time courses of all three oligomeric states (Figure 1k). The relatively low value of *k*_M→D_​  =  (3.7 ± 0.3) × 10^−3^ µM^−1 ^s^−1^ indicates slow dimerization, while the larger association rate *k*_D→T_  =  (75 ± 4) × 10^−3^ µM^−1 ^s^−1^ reflects a more efficient tetramerization step once dimers are present. The dissociation rates further reveal distinct stabilities: the dimer dissociates rapidly with *k*_D→M_​  =  (560 ± 70) × 10^−3^ s^−1^, whereas the tetramer is more stable (*k*_T→D_​  =  (6.3 ± 0.6) × 10^−3^ s^−1^). Together, these rate constants collectively explain the observed accumulation of tetramers and the persistence of monomers in a dynamic light‐induced stationary state. These results demonstrate that the experimentally observed oligomerization dynamics of CRY1 can be effectively described by a two‐step reversible reaction model, identifying dimer formation as the rate‐limiting step in CRY1 activation and providing quantitative insight into the kinetics of light‐induced assembly. In comparison, FAD photoreduction occurs within microseconds [38], much faster than oligomerization, indicating that the light‐induced activation of the flavin cofactor does not contribute any significant kinetic delay. The relative stabilities of the oligomers differ substantially, with dimers being highly transient while tetramers display significantly increased stability. These light‐dependent equilibria suggest that CRY1 signaling is a highly dynamic process rather than a simple on‐off switch. To directly compare our in vitro oligomerization kinetics with in vivo observations made by Liu et al. [22], we fitted the tetramer formation data using a single‐exponential model and determined a half‐life of approximately *t*_1/2_  =  18 s (Figure S4). In vivo, the corresponding half‐life at a fluence rate of 30 µmol m^−2 ^s^−1^ is about five times longer, reflecting additional cellular constraints such as regulatory interactions (e.g., BIC1 inhibition), metabolites, different reducing environment, temperature and, of course, different protein concentrations. Despite these differences, the timescales remain within the same order of magnitude, underscoring the physiological relevance of the oligomerization process captured by time‐resolved mass spectrometry. Importantly, our in vitro approach allows disentangling the influence of individual factors that cannot easily be resolved in the cellular context, thereby complementing in vivo measurements. While the kinetic parameters were determined for CRY1‐PHR, the underlying sequential monomer‐dimer‐tetramer assembly scheme is likely conserved among plant CRYs. In particular, the distinction between fast light‐induced oligomerization and slower thermal disassembly is expected to apply to CRY2 as well, consistent with reported similarities in photoreceptor regulation. Beyond this biological relevance, such a detailed kinetic analysis also opens opportunities for engineering CRY1‐based optogenetic tools with tunable temporal precision, complementing existing CRY2‐based optogenetic systems [39, 40, 41, 42, 43].

### 2.2 ATP Boosts CRY1 Oligomer Formation

Given previous reports that ATP influences the FAD photoreduction kinetics of CRYs [30, 32, 33, 34], we subsequently investigated whether ATP also affects the light‐induced oligomerization of CRY1. Under identical conditions in the presence and absence of ATP, we observed a pronounced ATP‐dependent enhancement of tetramer formation (Figure 1c,d). In the presence of 100 µM ATP, which is within the range of estimated physiological ATP concentrations in plant cells [44], CRY1 assembled into tetramers more efficiently and reached markedly higher tetramer levels upon blue light exposure (Figure 1d). Kinetic modeling shows that ATP modulates all association and dissociation steps of CRY1 oligomerization. While ATP accelerates monomer‐to‐dimer association by approximately two‐fold, it slows the reverse dimer dissociation by nearly an order of magnitude, resulting in pronounced stabilization of the dimeric intermediate (Figure 1m). Moreover, ATP enhances dimer‐to‐tetramer association by a factor of four, while slowing thermal tetramer dissociation by approximately twofold (Figures 1h–j,m and S3d–f). The combined effect of these changes shifts the stationary oligomer distribution toward the tetramer, rendering it the dominant species under continuous illumination (Figure 1l). To further assess the concentration dependence of this effect, we also tested a concentration of 200 µM ATP. Under these conditions, we detected a substantial increase in the dimeric CRY1 population already in the dark‐adapted state compared to samples without ATP or in the presence of 100 µM ATP (Figure S5a–c). This observation suggests that higher ATP concentrations promote or stabilize dimer formation even in the absence of blue light. These findings show that ATP enhances and stabilizes dimer formation, which we observed to be the rate‐limiting step for tetramer formation under blue light conditions, revealing a broader modulatory role of ATP in tuning CRY oligomerization dynamics. Absorption spectra also show the boosting effect of ATP on the kinetics of FAD photoreduction, as has already been shown in previous studies [30, 32, 33, 34]. In our measurements, the addition of ATP accelerated the photoreduction of oxidized flavin (FAD_ox_) to FADH° by approximately 12%, while slowing down the thermal decay of FADH° back to FAD_ox_ by nearly 60% (Figure S5d–h). While the acceleration of the photoreduction is small and might not be physiologically significant, our data overall support the notion that ATP acts as a cofactor, stabilizing the oligomeric state and promoting the conformational changes required for stable oligomer formation.

### 2.3 BIC1 Binds CRY1 in Both Darkness and Under Blue Light

**FIGURE 2:**
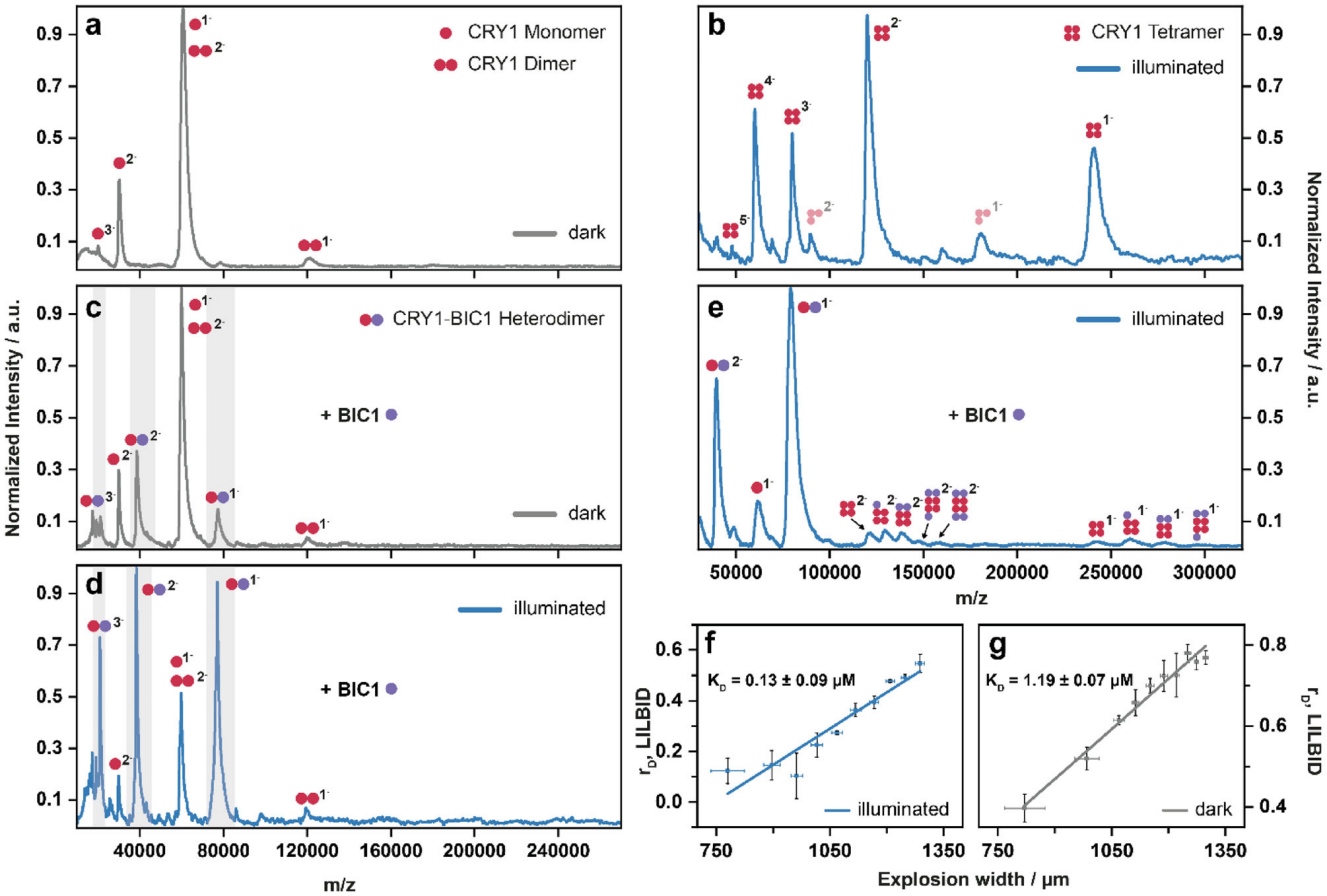
BIC1 inhibits CRY1 oligomerization in both dark and blue light conditions with higher affinity of BIC1 to CRY1 under blue light conditions. LILBID‐MS spectrum of CRY1 shows a monomer (pink dot; calculated molecular mass: 59.2 kDa, experimental molecular mass: 59.9 kDa) (a) in darkness, which forms tetramers upon illumination with blue light (b). CRY1 trimers are caused by laser dissociation. (c) Incubation of CRY1 monomer and BIC1 (purple dot, calculated molecular mass: 18.9 kDa, experimental molecular mass: 17.5 kDa) in an equimolar ratio in darkness results in a mixture of CRY1‐BIC1 heterodimer and CRY1 monomer. (d) Upon blue light exposure, the equilibrium shifts towards a higher proportion of the CRY1‐BIC1 heterodimer relative to the CRY1 monomer (highlighted in grey). (e) Under blue light conditions, addition of BIC1 to the CRY1 tetramer results in binding to each protomer, thereby dissociating the tetramer into CRY1‐BIC1 heterodimers. (f) qLILBID‐MS measurements reveal that the CRY1‐BIC1 heterodimer exhibits a 10‐fold higher affinity under blue light compared to its dark‐state counterpart (g). The annotated *K*_D_ values represent the mean of three independent measurements (*n* = 3).

While BIC1 is known to bind CRY1 [21, 25, 26, 27], it remains unresolved whether this interaction blocks CRY1 oligomerization or dismantles existing assemblies. We therefore directly investigated the mechanism of CRY1 inhibition by BIC1 using laser‐induced liquid bead ion desorption mass spectrometry (LILBID‐MS) [45]. To enable real‐time observation of light‐dependent effects, the LILBID‐MS setup was equipped with a blue‐light LED, which illuminated the sample throughout the measurements (Figure S1b). In line with the nESI‐MS data, LILBID‐MS analysis further confirmed that CRY1 exists predominantly as a monomer in the dark (∼60 kDa), with only a minor dimeric population (∼120 kDa) detectable (Figure 2a). Blue‐light illumination shifted the oligomeric distribution rapidly toward tetrameric assemblies (∼240 kDa), underscoring light‐dependent higher‐order organization of CRY1 (Figure 2b). A small trimeric CRY1 population was also detected, arising from partial dissociation of the tetramer induced by the energy input during LILBID‐MS analysis (Figure S6a). Upon addition of BIC1 (∼20 kDa) in both the dark and under blue light conditions, we observed a discrete charge distribution corresponding to the mass of CRY1‐BIC1 heterodimer (∼80 kDa), indicating direct binding between the two proteins (Figure 2c,d). While CRY1 exhibited reduced binding to BIC1 in the dark (Figure 2c), we observed a significant increase in CRY1‐BIC1 heterodimer formation under blue light conditions (Figure 2d). Notably, in light‐exposed samples, the CRY1 tetramer signal was absent, suggesting that BIC1 binding prevents oligomer formation. To quantify the interaction strength of CRY1‐BIC1 heterodimer in the dark and under blue light conditions, we employed quantitative LILBID‐MS (qLILBID‐MS), a label‐free technique capable of measuring dissociation constants under the required buffer conditions [46, 47, 48]. In the dark, BIC1 bound CRY1 with moderate affinity (*K*_D_ ≈ 1.2 µM) (Figure 2g), whereas under blue light, the interaction became more stable (*K*_D_ ≈ 0.13 µM) (Figure 2f). This 10‐fold increase in affinity suggests that light‐induced conformational changes in CRY1 enhance its recognition by BIC1. This demonstrates that BIC1 binds CRY1 in both its inactive and active states but with a strong preference for the light‐activated form, consistent with a mechanism in which BIC1 selectively inhibits photoactivated CRY1.

### 2.4 BIC1 Reverses CRY1 Oligomerization

Given that CRY1 tetramerizes upon blue light exposure (Figure 2b) and that BIC1 binds with high affinity under blue light (Figure 2d), the question arises whether BIC1 merely blocks tetramer formation or can even actively dismantle pre‐existing CRY1 oligomers, as was shown for CRY2 and BICs [21, 24]. Therefore, we added BIC1 to pre‐illuminated, tetrameric CRY1 under constant illumination with blue light. The mass spectra indicate that BIC1 binds sequentially to individual CRY1 protomers within the tetramer (∼260–320 kDa) (Figure 2e), displacing them from the tetrameric complex and forming a dominant BIC1–CRY1 heterodimer (∼80 kDa). These data suggest that BIC1 not only prevents tetramer formation but also actively dismantles existing CRY1 tetramers by occupying all subunits. Importantly, the direct observation of BIC1‐induced CRY dissociation by native mass spectrometry provides mechanistic insight at the molecular level and supports a general mode of BIC‐mediated inhibition in plant CRYs, extending previous observations reported for CRY2 using orthogonal approaches.

**FIGURE 3:**
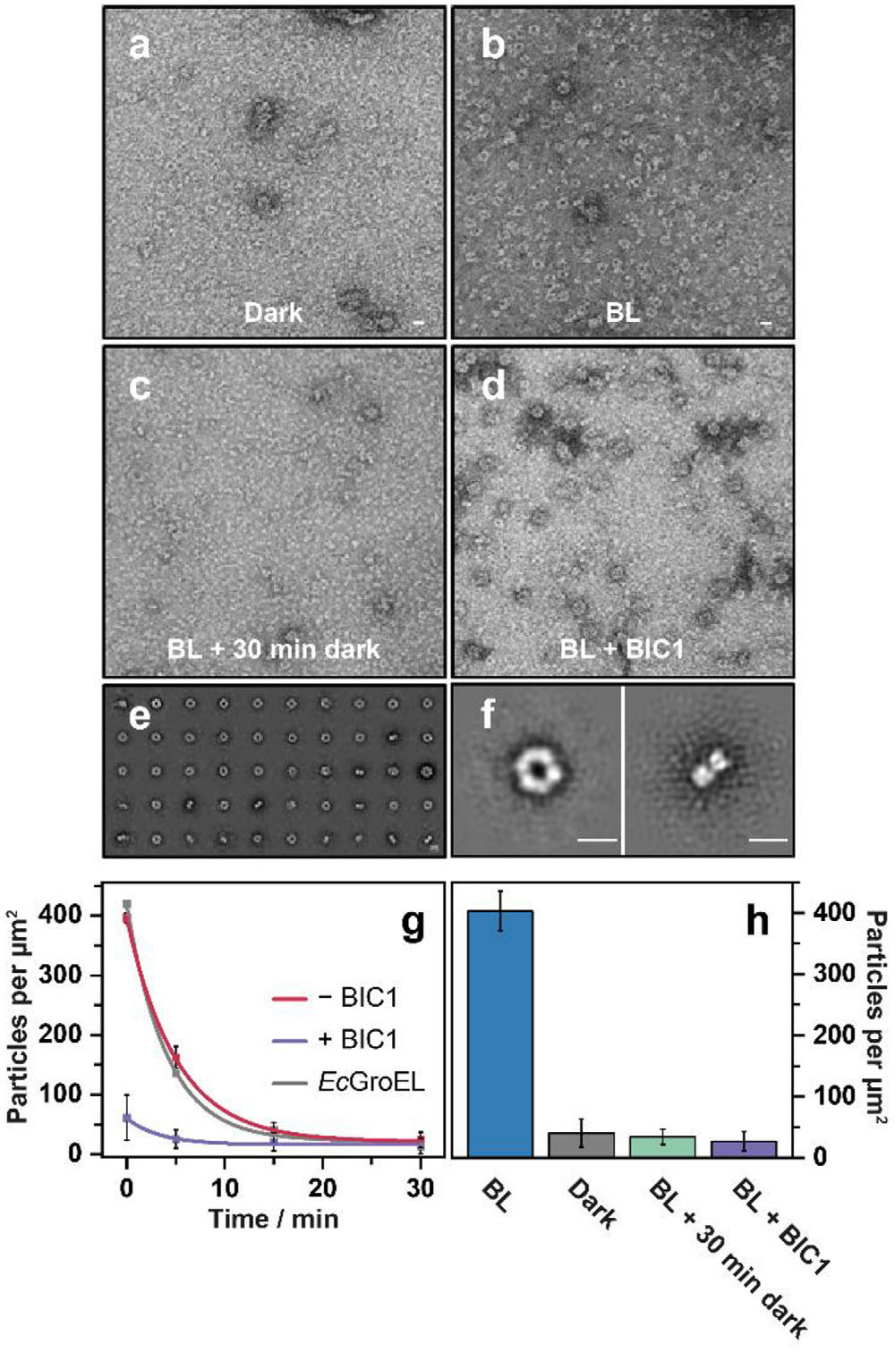
Blue‐light–induced ring formation and monomerization dynamics of CRY1. TEM images of negatively stained CRY1‐PHR samples show predominantly proteinaceous background in darkness (a), whereas blue‐light illumination induces the quantitative formation of doughnut‐shaped ring structures (b). (c) CRY1‐PHR after blue‐light exposure followed by 30 min incubation in darkness. (d) CRY1‐PHR under blue‐light illumination in the presence of BIC1. (e) Representative 2D class averages of blue‐light–induced CRY1‐PHR ring structures; 6734 particles were selected in total. (f) Top and side views of the donut‐shaped tetramer are shown, averaged from 176 and 150 particles, respectively. Scale bars, 10 nm. Blue‐light treatment was performed at 450–465 nm for 20 min with a fluence rate of 115 µmol m^−^^2^ s^−^^1^. (g) Kinetics of CRY1‐PHR monomerization following transfer from blue light to darkness in the absence or presence of BIC1 or *E. coli* GroEL as a negative control (*n*  =  3 ± SD). (h) Quantification of the effect of BIC1 on the decrease in the number of CRY1‐PHR particles, reflecting monomerization kinetics (*n*  =  3 ± SD).

Negative‐stain TEM corroborates the mass spectrometry data. In blue light‐treated samples, CRY1 formed doughnut‐like ring structures that were absent in dark‐adapted preparations (Figure 3a–c,e,f), consistent with the formation of tetrameric assemblies. The addition of BIC1 markedly reduced the abundance of these oligomeric particles. Even immediately after 30 min of illumination, CRY1 samples incubated with BIC1 displayed few ring‐like structures (Figure 3d,h). Moreover, following transfer to darkness, CRY1 oligomers disassembled significantly faster in the presence of BIC1, with near‐complete monomerization observed at the earliest time point (Figure 3g).

### 2.5 BIC1 Disassembles CRY1 Tetramers Independently of Light Activation

**FIGURE 4:**
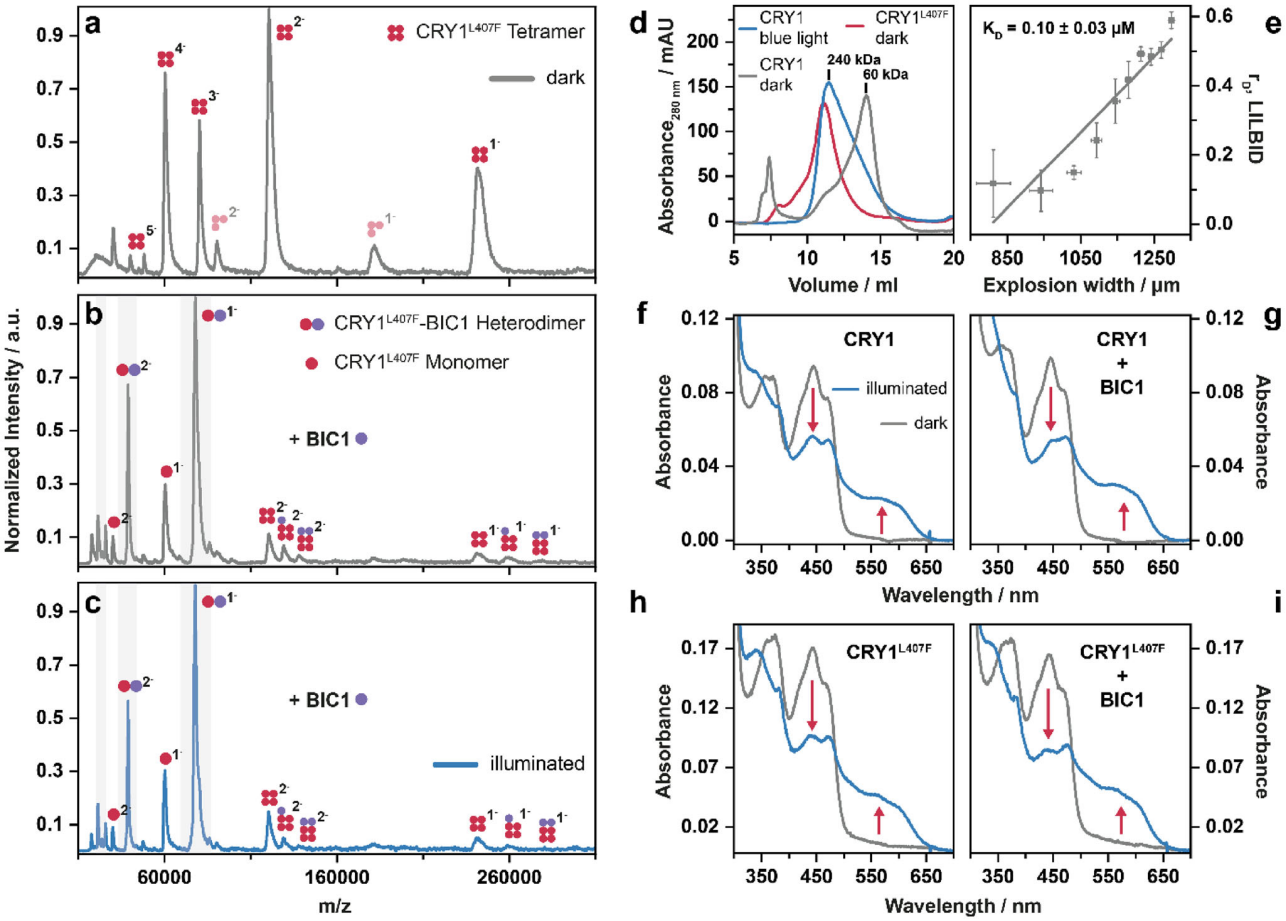
BIC1 disassembles constitutively active CRY1^L407F^ tetramers and does not inhibit photoreduction of the FAD cofactor. (a) LILBID‐MS spectrum of the constitutively active CRY1^L407F^ mutant shows a tetramer in darkness (calculated molecular mass: 236.8 kDa, experimental molecular mass: 241.4 kDa). CRY1^L407F^ trimers are caused by laser dissociation. (b) Addition of BIC1 (purple dot) to CRY1^L407F^ in an equimolar ratio in darkness results in binding to each protomer, thereby dissociating the CRY1^L407F^ tetramer into CRY1^L407F^‐BIC1 heterodimer and monomeric CRY1^L407F^. (c) Exposure to blue light does not shift the equilibrium toward the CRY1^L407F^‐BIC1 heterodimer, as has been shown for wild‐type CRY1. (d) SEC elution profiles of wild‐type CRY1 kept in darkness or irradiated with blue light, and of hyperactive CRY1^L407F^ mutant kept in darkness. The broadened elution profile of CRY1 in the dark state could indicate a certain proportion of oligomeric states already under these experimental conditions. (e) qLILBID‐MS measurement of the CRY1^L407F^‐BIC1 heterodimer under dark conditions revealed that BIC1 binds CRY1^L407F^ with high affinity (*n* = 3). (f) Absorption spectra of wild‐type CRY1 before (grey line) and after (blue line) blue light exposure indicate FAD photoreduction, with a decrease in FAD_ox_ peak at 450 nm, and the emergence of a broad band at 550–600 nm, characteristic for the FADH° semiquinone. (g) Addition of BIC1 to wild‐type CRY1 does not inhibit the photoreduction of CRY's FAD chromophore. (h) Absorption spectra of the CRY1^L407F^ mutant indicate that chromophore photoreduction remains unaffected by the mutation. (i) Photoreduction of the FAD chromophore in the CRY1^L407F^ mutant remains unaffected by BIC1 addition, as observed for wild‐type CRY1.

To examine whether BIC1‐mediated inhibition of CRY1 tetramers requires light activation, we analyzed the CRY1^L407F^ mutant, which has been observed to be hyperactive and results in a constitutive photomorphogenic phenotype [36, 37]. Analytical size exclusion chromatography (SEC) revealed that CRY1^L407F^ already shows a tetrameric retention time in darkness compared to the wild‐type CRY1 (Figure 4d). LILBID‐MS confirmed the presence of tetramers for CRY1^L407F^ in darkness (Figure 4a), and absorption spectroscopy has shown that the leucine to phenylalanine replacement at position 407 does not impair photoreduction of the chromophore (Figure 4h).

Upon addition of BIC1, the CRY1^L407F^ tetramers were efficiently disassembled into BIC1–CRY1^L407F^ heterodimers (Figures 4b and S6b). In contrast to the wild‐type CRY1 (Figure 2c,d), exposure to blue light did not further enhance the formation of the CRY1^L407F^‐BIC1 heterodimer (Figure 4c) or have any other significant effect on the observed complexes. qLILBID measurements revealed that BIC1 binds CRY1^L407F^ independently of illumination with high affinity (*K*_D_  =  0.10 ± 0.03 µM), comparable to the affinity of BIC1 for light‐activated wild‐type CRY1 (*K*_D_  =  0.13 ± 0.09 µM). This result demonstrates that the L407F mutation induces a conformational change in CRY1 that mimics its light‐activated state with respect to BIC1 recognition. Collectively, these findings suggest that BIC1 disrupts CRY1 oligomerization by binding individual CRY1 protomers with higher affinity than the protomer‐protomer interactions, effectively outcompeting CRY1‐CRY1 contacts regardless of the photoreduction state of the chromophore.

### 2.6 BIC1 Does Not Affect the Photoreduction Kinetics of the CRY1 FAD Cofactor

The remaining open question is whether BIC1 suppresses CRY1 oligomerization by interfering with its photoactivation, as previously demonstrated for CRY2 inhibition by BIC2 [24]. To address this, we examined the photoreduction state of the FAD chromophore in the presence and absence of BIC1. Absorption spectra of purified CRY1 recorded before and after blue light exposure showed the expected spectral changes associated with FAD photoreduction (Figures 4f and S5d,e). In the dark‐adapted state, CRY1 displayed absorption maxima characteristic of FAD_ox_ at 450 nm and a shoulder near 480 nm. Upon blue light illumination, these peaks decreased in intensity, accompanied by the appearance of a broad band between 550 and 600 nm, consistent with the formation of the neutral semiquinone radical (FADH°) [17, 18, 49]. These spectral changes are characteristic for the light‐driven redox transition of the CRY1 chromophore. Identical spectral features were observed when CRY1 or CRY1^L407F^ were incubated with BIC1 under the same conditions (Figures 4g,i and S7a–c), indicating that BIC1 does not impair the light‐induced redox transition of the FAD_ox_ to FADH°. This suggests that BIC1 does not affect the initial photoactivation step of CRY1. Instead, it acts downstream of photoreduction, targeting the light‐induced oligomeric states of CRY1. These findings indicate that the inhibitory mechanism of CRY1 differs from that of CRY2, supporting the idea that while CRYs share a conserved photochemical activation step, their downstream regulation can vary, allowing for functional specificity.

## 3 Conclusion

**FIGURE 5:**
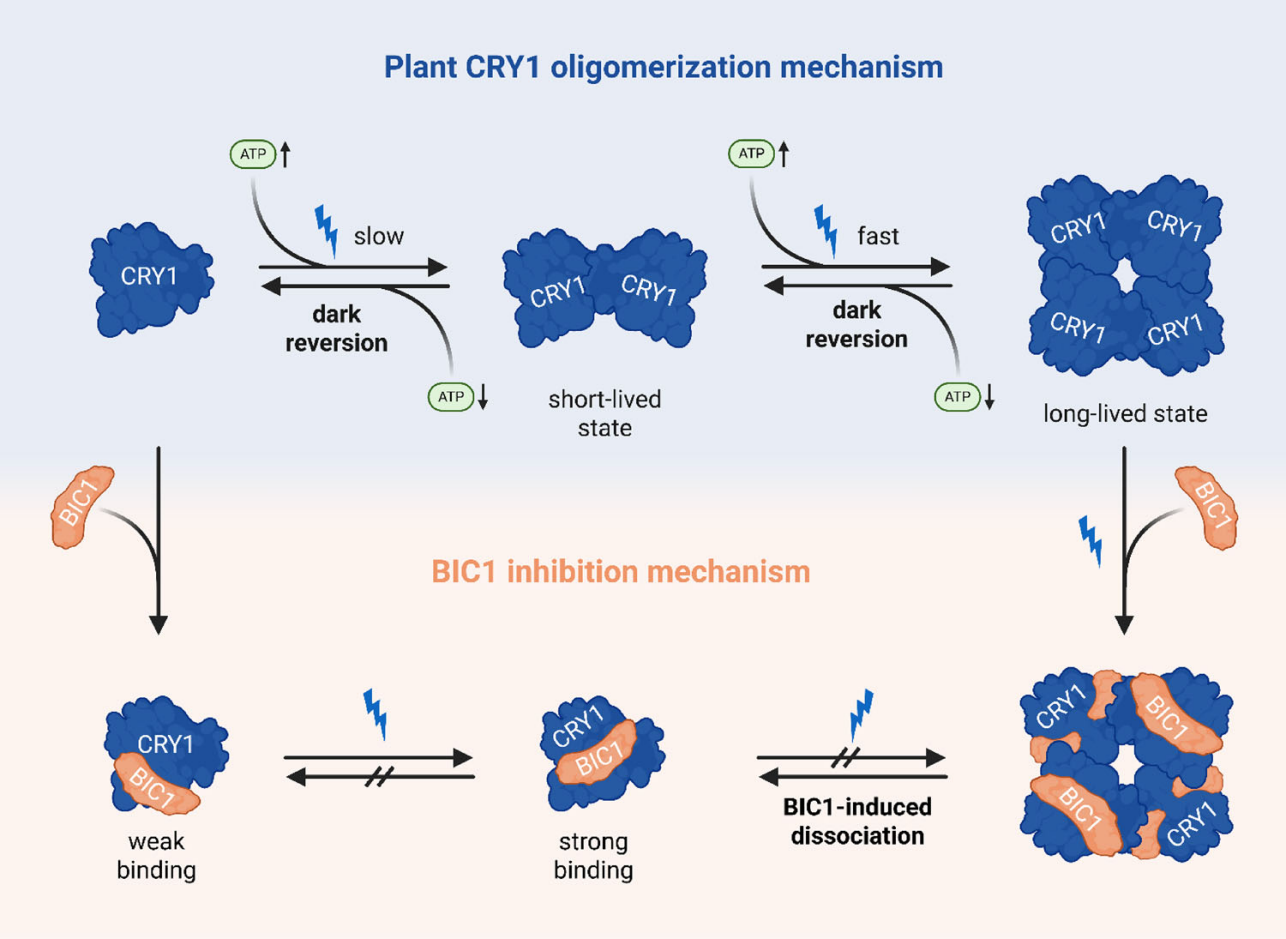
Schematic model of plant CRY oligomerization dynamics, ATP modulation, and BIC1‐mediated inhibition. Upon blue light exposure, CRY1 transitions from a monomeric to a tetrameric state via a short‐lived dimeric intermediate. This oligomerization follows a sequential, reversible two‐step mechanism. ATP enhances the association rates for dimer and tetramer formation, while slowing down the dissociation rates of these oligomers. BIC1 binds CRY1 in both dark and light conditions, with significantly higher affinity for the light‐activated conformation. This interaction prevents oligomer formation and actively disassembles pre‐formed tetramers by binding to individual CRY1 protomers, measured under continuous blue‐light illumination to exclude thermal dissociation. Figure was created with Biorender.com.

Light perception in plants is a dynamic process influenced by multiple molecular factors. Our kinetic analysis of plant CRY1 reveals that oligomerization proceeds through discrete assembly steps and is modulated by both metabolic signals and inhibitory interactions. These findings suggest that CRY1 activity can be finely adjusted in response to changing conditions, enabling a more nuanced regulation of light signaling. Time‐resolved native mass spectrometry establishes a clear kinetic pathway for plant CRY oligomerization, exemplified here for *Arabidopsis* CRY1 (Figure 5). The sequential monomer‐dimer‐tetramer assembly is consistent with a two‐step reversible kinetic model. Beyond intrinsic kinetics, our findings highlight how ATP modulates CRY1 activity by stabilizing oligomeric intermediates and promoting higher‐order assemblies, shifting the equilibrium toward tetramers. This is particularly intriguing given that ATP is not only a fundamental energy currency but also binds the PHR domain of plant CRYs [21, 28] and influences FAD photoreduction kinetics [30, 32, 33, 34].

Our data further elucidate the inhibitory role of BIC1, revealing an elegant regulatory mechanism (Figure 5). BIC1 binds to CRY1 in both dark and light conditions, with enhanced affinity for the light‐activated form. This light‐dependent increase in affinity provides a mechanism for selective inhibition of active CRY1. Moreover, we provide clear evidence that BIC1 is an active disassembler of CRY1 oligomers, rather than just a passive competitor. The strong interaction of BIC1 with the constitutively active CRY1^L407F^ mutant, which mimics the tetrameric light‐activated state, indicates recognition of a specific conformational form independent of FAD redox state. Notably, BIC1 does not interfere with FAD photoreduction, differing from recent reports on the CRY2–BIC2 interaction [24]. In summary, our work establishes a quantitative kinetic model for CRY1 oligomerization. By showing that ATP acts as an oligomerization accelerator and BIC1 as an active disassembler, we provide a more detailed and nuanced understanding of how CRY1 activity is regulated beyond simple light perception. These insights not only shed light on the functional specialization of CRY1 but also highlight a general principle of plant signaling, where a photoreceptor's output is shaped by a complex interplay between light, metabolites, and dedicated regulatory proteins.

## Conflicts of Interest

The authors declare no conflicts of interest.

## Supporting information



The authors have cited additional references within the Supporting Information [37, 45, 46, 47, 48, 50, 51, 52].**Supporting File 1**: Anie72073‐sup‐0001‐SuppMat.Docx.

## Data Availability

The data that support the findings of this study are available from the corresponding author upon reasonable request.
