# Supplementary material for: Time‐Resolved Native Mass Spectrometry Reveals Reversible Light‐Driven Oligomerization of Arabidopsis Cryptochrome 1 and Its Antagonism by BIC1
Source: Angew Chem Int Ed Engl. 2026 Apr 7;65(21):e25792. doi: 10.1002/anie.202525792 (PMC13182210; doi:10.1002/anie.202525792)
Supplement: Supplementary file 1 — The authors have cited additional references within the Supporting Information [37, 45, 46, 47, 48, 50, 51, 52].Supporting File 1: Anie72073‐sup‐0001‐SuppMat.Docx. [file ANIE-65-e25792-s001.docx]

**Supporting Information**

**Time-Resolved Native Mass Spectrometry Reveals Reversible Light-Driven Oligomerization of *Arabidopsis* Cryptochrome 1 and its Antagonism by BIC1**

Alicia Just^[a]^, Nils Niemann^[b]^, Petra Gnau^[c]^, Dennis Kock^[b]^, Thomas Heimerl^[d]^, Stephan Kiontke^[b,d]^, Lars-Oliver Essen^[c]^, Alfred Batschauer^[b]^ and Nina Morgner*^[a]^

[a] A. Just, Prof. Dr. N. Morgner
Institute of Physical and Theoretical Chemistry
Johann Wolfgang Goethe University
Max-von-Laue-Straße 9, 60438 Frankfurt am Main, Germany
E-mail: morgner@chemie.uni-frankfurt.de

[b] Dr. N. Niemann, Dr. D. Kock, Dr. S. Kiontke, Prof. Dr. A. Batschauer
Department of Biology
Philipps University
Karl-von-Frisch-Straße 8, 35043 Marburg, Germany

[c] P. Gnau, Prof. Dr. L.-O. Essen
Department of Chemistry
Philipps University
Hans-Meerwein-Straße 4, 35032 Marburg, Germany

[d] Dr. T. Heimerl, Dr. S. Kiontke
SYNMIKRO Research Center and Department of Chemistry
Philipps University
Karl-von-Frisch-Straße 14, 35032 Marburg, Germany

Table of Contents

[Methods 1](#_Toc213764818)

[Cloning of Constructs 1](#_Toc213764819)

[Overproduction and Purification of Recombinant Proteins 1](#_Toc213764820)

[Size Exclusion Chromatography (SEC) 2](#_Toc213764821)

[Photoreduction and Dark Reversion of CRY1 2](#_Toc213764822)

[Nano Electrospray Ionization Mass Spectrometry 2](#_Toc213764823)

[nESI-MS based Rate Constant Determination 2](#_Toc213764824)

[LILBID-MS 3](#_Toc213764825)

[TEM Negative Staining 4](#_Toc213764826)

[Figures 5](#_Toc213764827)

# Methods

## Cloning of Constructs

The expression vectors encoding the PHR domain (residues 1–509) of *Arabidopsis thaliana* CRY1 and the CRY1^L407F^ mutant, both cloned into the pACYCDuet-1 vector have been described previously.^[37]^ To prevent disulfide bond-mediated dimerization observed in the *Arabidopsis thaliana* wild-type BIC1 protein (SI Figure S8a, b), a synthetic gene encoding a BIC1 variant carrying three cysteine-to-alanine substitutions (C99A, C105A, C136A) was generated. This triple mutant, named BIC1 throughout the text, was synthesized and cloned into the pET28a(+) vector (*BioCat GmbH*). Recombinant proteins carried an N-terminal His₆-tag.

## Overproduction and Purification of Recombinant Proteins

Expression and purification of CRY1 and the CRY1^L407F^ mutant were adapted from a previously described protocol^[37]^ with minor modifications and yielded protein samples of comparable quality to those shown in Fig. 1 of the previous study. Briefly, for expression of CRY1 and the CRY1^L407F^ mutant as well as BIC1^3CA^, competent *E. coli* BL21 Star (DE3) cells (*Thermo Fisher*) were used. For CRY1 and CRY1^L407F^ overproduction, 20 mL overnight cultures were inoculated into 2 L LB medium supplemented with 34 µg/mL chloramphenicol in 5 L baffled flasks. Cultures were incubated at 37 °C with shaking (110 rpm) until an OD_600 nm_ of ~1.0 was reached. Cells were placed on ice for 30 min, and expression was induced with 0.25 mM IPTG. All further steps were carried out under red light. Cultures were incubated for 3 days at 16 °C. Cells were harvested by centrifugation (5,330g, 15 min, 4 °C) and resuspended in lysis buffer (50 mM MOPS, pH 7.4, 300 mM NaCl, 10% glycerol, 1% Triton X-100). Lysozyme (0.2 mg/mL), PMSF (0.4 mM) and DNase I were added, and the suspensions were incubated for 10 min at room temperature before disruption using a French press. Lysates were clarified by centrifugation (39,000g, 30 min) and filtered. The supernatant was applied onto a 1 mL Protino Ni-NTA column (*Macherey-Nagel*), followed by sequential washing steps with lysis buffer, buffer containing 3% Triton X-100, and lysis buffer without TX-100 until a stable UV baseline was reached. Non-specifically bound proteins were removed with 10% elution buffer (50 mM MOPS, pH 7.4, 300 mM NaCl, 10% glycerol, 250 mM imidazole). Recombinant CRYs were eluted with 100% elution buffer. CRY1 concentrations were kept below 1 mg/mL to prevent oligomerization. Final purification was performed by size-exclusion chromatography (Superdex 200 pg 16/60, *Cytiva*).

BIC1 purification followed a similar protocol. Expression was induced with 0.5 mM IPTG, and cultures were incubated for 18 h at 25 °C. Cells were harvested, washed with PBS, and resuspended in lysis buffer (50 mM HEPES, pH 7.0, 500 mM NaCl, 10% glycerol). Cell disruption was performed as described above, using a protease inhibitor mix (*Carl Roth, Cat. no. 3760.1*) in place of PMSF. The soluble fraction was loaded onto a Ni-NTA column and washed with protein buffer (50 mM HEPES, pH 7.0, 300 mM NaCl, 10% glycerol), followed by a wash containing 3% Triton X-100 and protein buffer. Additional washes were performed with 10% and 20% elution buffer (50 mM HEPES, pH 7.0, 300 mM NaCl, 250 mM imidazole, 10% glycerol). BIC1 was eluted with 100% elution buffer, concentrated, and further purified via size-exclusion chromatography (Superdex 75 pg 16/60, *Cytiva*).

## Size Exclusion Chromatography (SEC)

For analysis of blue light-dependent oligomerization, samples containing 30 μM CRY1 and 10 mM ß-mercaptoethanol in 100 µl volumes were separated on a Superdex 200 10/300 GL column (*GE Healthcare*) equilibrated at 10 °C in buffer D containing 20 mM ß-mercaptoethanol. CRY1 was pre-illuminated with blue light (465 ± 5 nm, 115 µmol m^−2^s^−1^) for 20 min. Three blue light LEDs were used to illuminate the column resin. Dark controls were kept in red safe light. Calibration of the column was done using gel-filtration standards (*Sigma Aldrich*).

## Photoreduction and Dark Reversion of CRY1

Absorbance changes of bound flavin were measured by UV-Vis spectroscopy (*Specord 600, Analytik Jena*). Before photoreduction, the CRY1 solution was centrifuged (10 min, 16873 g, 4 °C) to pellet and remove aggregated protein. A protein solution of 10 µM CRY1 was prepared, and dithiothreitol (DTT) added to a final concentration of 10 mM. Where indicated, BIC1 was added at an equimolar concentration (10 µM). For photoreduction, samples were irradiated with a 455 nm LED (M455L2, *Thorlabs*) at 100 µmol m^−2^s^−1^. At defined time points, the absorption between 200 nm – 900 nm was recorded. The dark conversion was monitored with the same photoreduced sample by leaving it in the dark until full recovery and absorbance recording. During all steps, cuvettes were chilled at 20 °C.

## Nano Electrospray Ionization Mass Spectrometry

nESI-MS was carried out using a Synapt G2-S instrument (*Waters Corporation, Wilmslow, UK*) equipped with a high-mass quadrupole modification. Nano electrospray emitters are produced in-house by pulling borosilicate glass capillaries with a Flaming/Brown micropipette puller (P-1000, *Sutter Instrument Co.*) and subsequently coated with a gold layer via sputtering. CRY1 (15 µM) was analyzed at room temperature in positive ion mode under a capillary voltage of 1.1 kV. The ion source was operated with a cone voltage of 150 V, an offset of 100 V, and maintained at 20 °C. The scan time was set to 1 scan per second. Calibration is performed using a standard cesium iodide (CsI) solution. Sample illumination was carried out by irradiation of the nano electrospray emitters with a 455 nm LED (M455L4, *Thorlabs*) at 30-50 µmol m^−2^s^−1^ (SI Figure S1a), using an illumination setup like that described by Camacho *et al*. (2019).^[50]^ Acquisition and data processing are achieved using MassLynx™ V4.1 SCN901. Prior to measurements, CRY1 was buffer-exchanged in 200 mM ammonium acetate, 10 mM DTT and pH 7.4, using Zeba Micro Spinbuffer exchangers (7 kDa MWCO, *Thermo Fisher Scientific*).

## nESI-MS based Rate Constant Determination

The measurements of the rate constants were performed following the same procedure as for the nESI-MS measurements, using a total CRY1 concentration of 15 µM, except for the scan time, which was set to 10 scans per second. The intensity (counts) of each species (monomer, dimer, tetramer) during the measurement period was estimated by generating an extracted ion chromatogram using MassLynx™ V4.1 SCN901. Initial concentrations of each species (monomer, dimer, tetramer) were determined from the measured signal intensities, assuming a total sample concentration of 15 µM relative to the molecular mass of the monomer. Kinetic data were analyzed by using DynaFit^[51]^ software and a reversible two-step kinetic model (Figure 1l) based on the differential equations 1-3. During the fitting, the measured intensities of dimers and tetramers were corrected using response coefficients to account for their lower signal due to higher stoichiometry.

|  | $\frac{ⅆ\left[ M \right]}{ⅆt}=-2k_{M\to D}\left[ M \right]\left[ M \right]+2k_{D\to M}\left[ D \right]$ | (1) |
| --- | --- | --- |
|  | $\frac{ⅆ\left[ D \right]}{ⅆt}=+k_{M\to D}\left[ M \right]\left[ M \right]-k_{D\to M}\left[ D \right]-2k_{D\to T}\left[ D \right]\left[ D \right]+2k_{T\to D}\left[ T \right]$ | (2) |
|  | $\frac{ⅆ\left[ T \right]}{ⅆt}=+k_{D\to T}\left[ D \right]\left[ D \right]-k_{T\to D}\left[ T \right]$ | (3) |

## LILBID-MS

Prior to analysis by LILBID-MS^[45]^, CRY1 and BIC1^3CA^ were buffer-exchanged as described for nESI-MS. All measurements were performed at 20 °C, using 4 μL of buffer-exchanged and degassed sample containing 15 µM CRY1 (monomer equivalent). For incubation experiments with BIC1^3CA^, CRY1 and BIC1^3CA^ were mixed and analyzed at equimolar concentrations (15 µM each). Microdroplets with a diameter of 50 μm are generated using a piezo-driven droplet generator (MD-K-130, *Microdrop Technologies GmbH,* Norderstedt, Germany) operating at 10 Hz. The droplets are transferred into a vacuum of about 10^-5^ mbar and irradiated with a pulsed infrared laser (*Innolas Spitlight 400, Continuum PowerLite 8000*) tuned to the vibrational absorption maximum of water (2.8 μm). Absorption of laser energy (10-23 mJ per pulse) by water molecules causes an explosive expansion of the droplets, resulting in the release of analyte ions into the gas phase. The ions are accelerated using a pulsed electric field and analyzed with a home-built time-of-flight mass spectrometer in negative ion mode. Each mass spectrum represents an average signal from 1,000 individual microdroplets. Mass spectra are calibrated against a 10 μM aqueous bovine serum albumin standard and subsequently processed by smoothing and background subtraction. The sample was exposed to blue light (455 nm LED, M455L4, *Thorlabs*) at 100 µmol m^−2^s^−1^ inside the droplet generator via a coupled optical fibre that was installed on the generator (SI Figure S1b).

For qLILBID-MS^[46,47,48]^ experiments the laser energy transferred into the droplets is correlated to the degree of complex dissociation. Therefore, the explosion of the irradiated droplets is visualized by illuminating the explosion with a green laser (Minilite I, *Continuum*, San Jose, USA) and the resulting images are captured using a high-speed camera (DFK 23UP031, *Imaging Source*, Bremen, Germany). Each individual droplet yields both a mass spectrum and a corresponding image of the explosion. The explosion width of each droplet, measured 5 µs after irradiation, is extracted from the recorded images using OpenCV and a custom-written Python script and serves as a proxy for the effective laser energy absorbed by the droplet. Increasing explosion widths correspond to increasing degrees of laser-induced dissociation of non-covalent complexes. For each droplet, the relative dissociation fraction is quantified from the corresponding mass spectrum (equation 4) as described by Schulte et al. (2025)^[48]^.

| $rD,LILBID=\frac{\int Monomer peaks}{\int Monomer peaks+\int Dimer peaks}$ | (4) |
| --- | --- |

Peak integrals are determined from the time-of-flight spectra. Plotting r_D_,LILBID as a function of explosion width yields dissociation curves that directly relate energy transfer to complex stability. K_D_ values are derived from these dissociation curves by comparison to calibration standards with known affinities measured under identical experimental conditions, as established previously^[46,47,48]^.

## TEM Negative Staining

Carbon coated copper grids (400 mesh) were hydrophilized by glow discharging (*PELCO easiGlow*, Ted Pella, USA). 5 µl of a protein suspension with a concentration of 30 µg/ml was applied onto the hydrophilized grids blotted with filter paper, washed with a droplet of sterile filtered H_2_O_bidest_, blotted again and stained with 2% (w/v) uranyl acetate. If applicable, the whole staining procedure was done under blue (465 ± 5 nm, 115 µmol m^−2^s^−1^) or safe red light, respectively. Samples were analyzed with a JEOL JEM-2100 transmission electron microscope using an acceleration voltage of 120 kV. For image acquisition a F214 FastScan CCD camera and EMMenu4 software (*TVIPS, Gauting*) was used.

To elucidate the influence of BIC1 on CRY1 oligomerization, CRY1 samples were pre-irradiated with blue light as described above, and the resulting donut-like assemblies were quantified via TEM. Ring structures with a diameter of 10 nm were counted manually within a defined area of 1 µm^2^. To account for potential spatial fluctuations and ensure statistical significance, the particle distribution was verified as approximately homogeneous across the TEM grid, and all experiments were performed as technical triplicates. Under blue-light conditions, a density of approximately 400 particles per 1 µm^2^ was observed. Since we observed chaperones in the BIC1 sample from the heterologous overexpression in E. *coli*, we included *Ec*GroEL as a control in the kinetics shown.

To emphasize the donut shaped nature of the CRY1 tetramers a 2D class averaging was done. For this 242 single 2k pictures were taken at a nominal magnification of 60kx with a pixel size of 0.16 nm. In cisTEM^[52]^ 6734 particles were picked and averaged to 50 2D classes.

# Figures




**Figure S1.** LED-based illumination setups for nESI-MS and LILBID-MS. a) Schematic representation of the LED-based illumination setup for nESI-MS. A 445 nm LED is directed onto the sample-filled capillary via a collimator, allowing illumination of the electrospray emitter at the front end of the mass spectrometer. b) Schematic representation of the integrated light source used in the LILBID mass spectrometer. Light from a 455 nm LED is coupled into an optical fibre via an adjustable collimator and lens system and directed onto the droplet generator within the vacuum chamber.


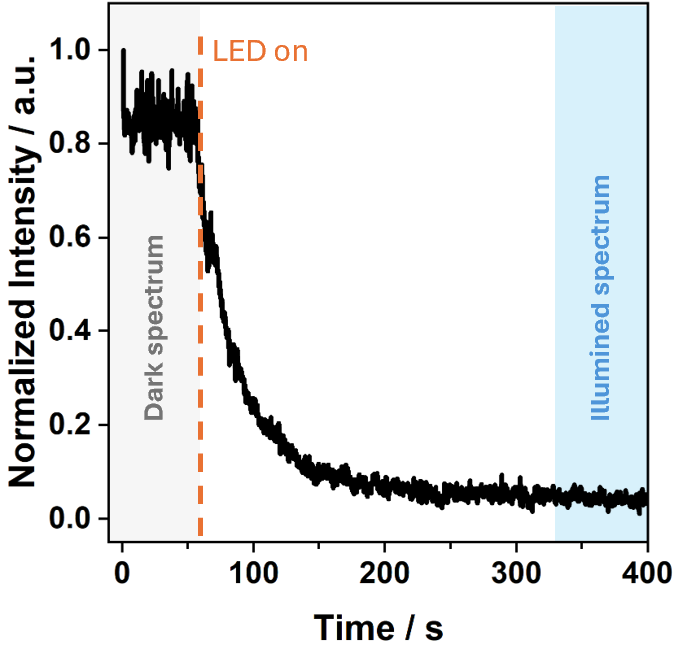

**Figure S2:** Representative monomer chromatogram illustrating timing of blue-light illumination relative to kinetic measurements. Spectra corresponding to Figure 1a and c were recorded during 60 s in the dark before LED activation (t₀). Spectra corresponding to Figure 1b and d were recorded under continuous blue-light illumination after the system reached steady state. The time of LED activation is indicated by a vertical dashed line.


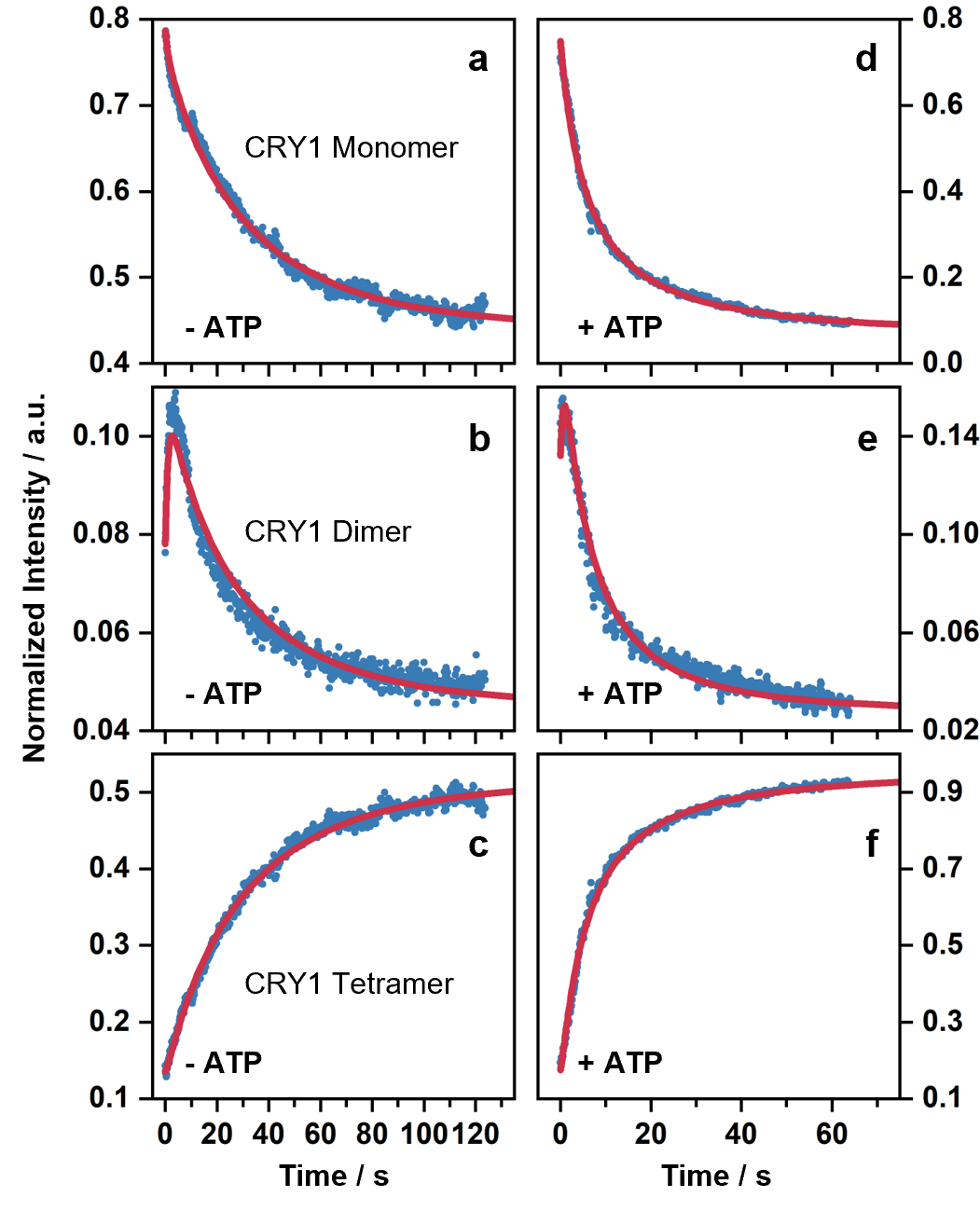


**Figure S3**: Oligomerization kinetics of CRY1-PHR under blue light and the influence of ATP plotted on a linear time scale. Time-resolved mass spectrometry measurements of the CRY1 monomer (a), dimer (b) and tetramer (c) in absence and presence of 100 µM ATP (d-f) and the fit (pink line) obtained from the two-step reversible kinetic model.


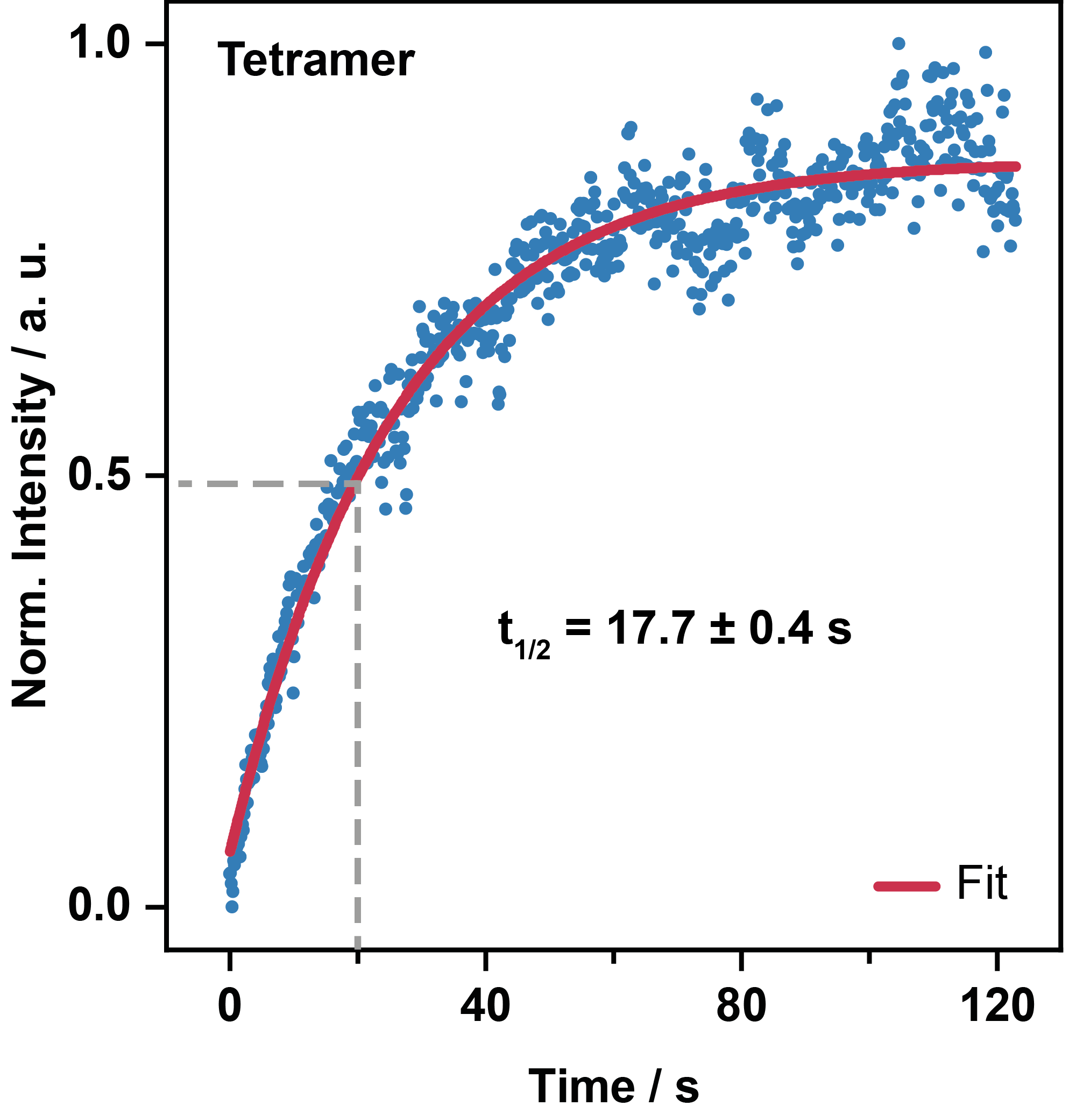


**Figure S4.** Light-dependent tetramer formation of CRY1 obtained from time-resolved nESI-MS. Fitting the data using a single-exponential model, yielding a half-life of t_1/2_ = 17.7 ± 0.4 seconds.


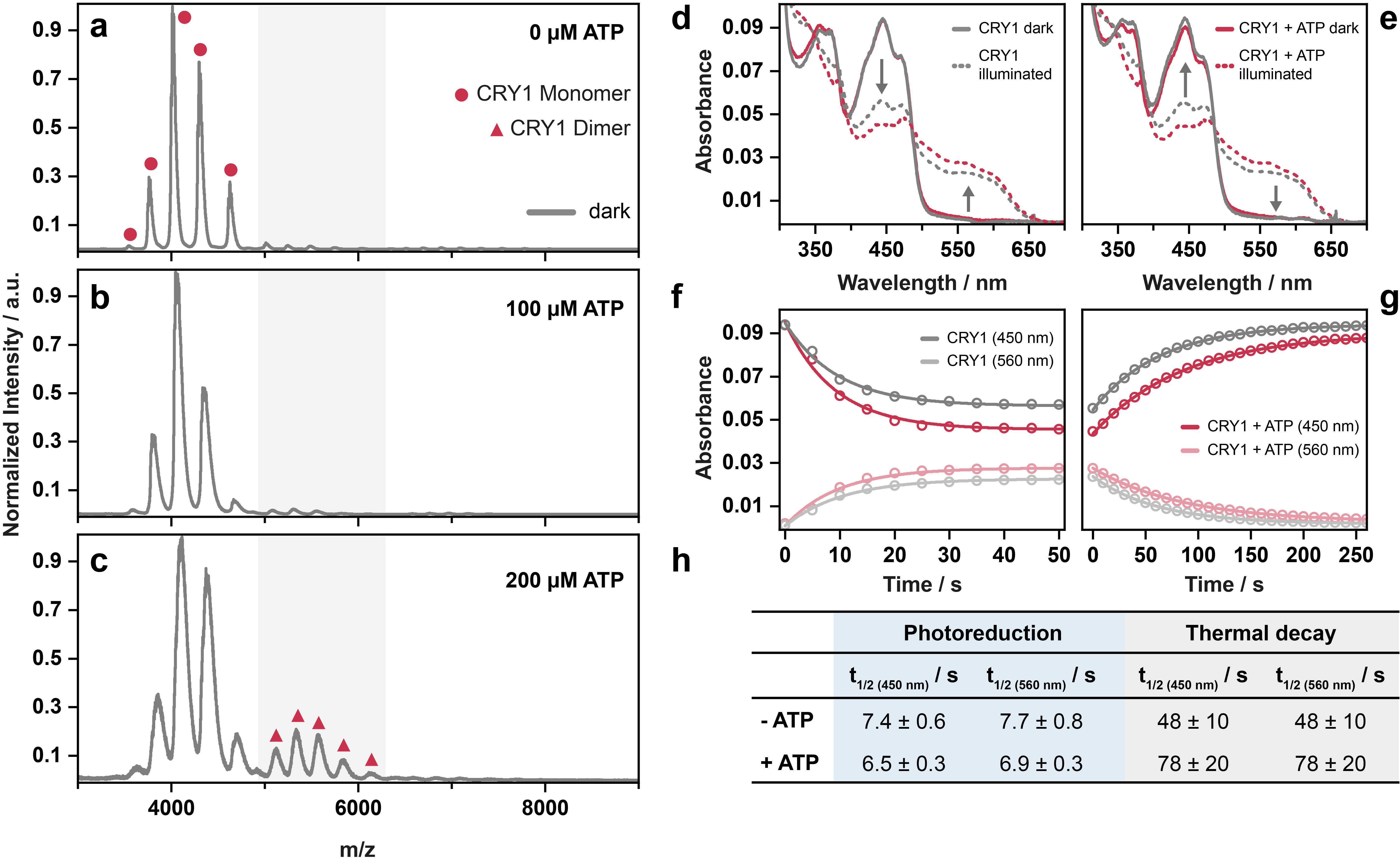


**Figure S5.** ATP modulates CRY1 dimerization in darkness and enhances FAD photoreduction kinetics. a-c) nESI-MS spectra of CRY1 in the absence of blue light at different ATP concentrations. Incubation with 200 µM ATP resulted in an increase of dimeric CRY1 species (c) compared to samples with 0 (a) or 100 µM ATP (b). d-h) ATP accelerates photoreduction and stabilizes the reduced state of the FAD cofactor; panels d-g show representative traces from a single measurement. Addition of 100 µM ATP enhanced the rate of FAD_ox_ to FADH° photoreduction by ~12% (f, h) and slowed the thermal reoxidation of FADH° to FAD_ox_ by ~60% (g, h); the corresponding rates are from three independent measurements (n = 3 ± SD).





**Figure S6.** LILBID-MS spectra showing dissociation of the CRY1 tetramer into a trimer upon increasing laser energy and time-dependent disassembly of CRY1^L407F^ tetramers by BIC1. a) Increasing laser energy results in a decrease of the CRY1 tetramer signal (highlighted in dark red) and the appearance of a trimeric species (highlighted in light red), indicating partial dissociation of the complex. b) BIC1 induces a progressive disassembly of CRY1^L407F^ tetramers in darkness with increasing incubation time by binding to individual CRY1^L407F^ protomers.


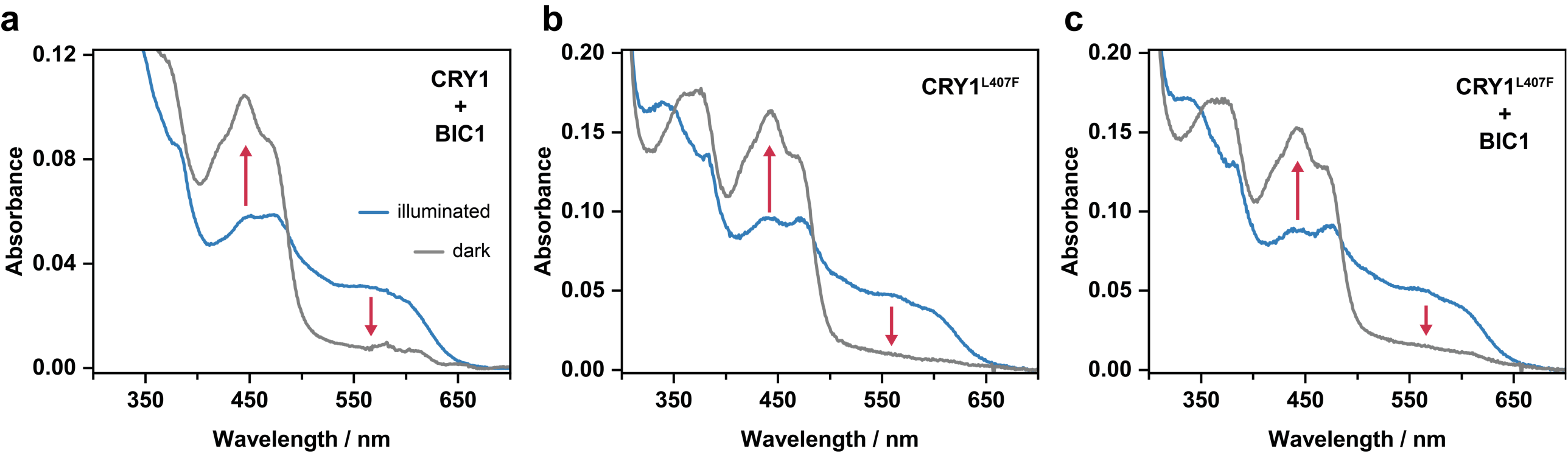


**Figure S7.** Absorption spectra slowing the thermal reoxidation of the FADH° semiquinone to FAD_ox_ in wild-type CRY1 and CRY1^L407F^ mutant incubated with BIC1. a) Thermal reoxidation of the reduced FAD chromophore of CRY1 incubated with BIC1. b-c) Thermal reoxidation of the reduced FAD chromophore of CRY1^L407F^ showing that the L to F replacement at position 407 and incubation with BIC1 in an equimolar ratio does not impair the chromophore’s thermal relaxation.


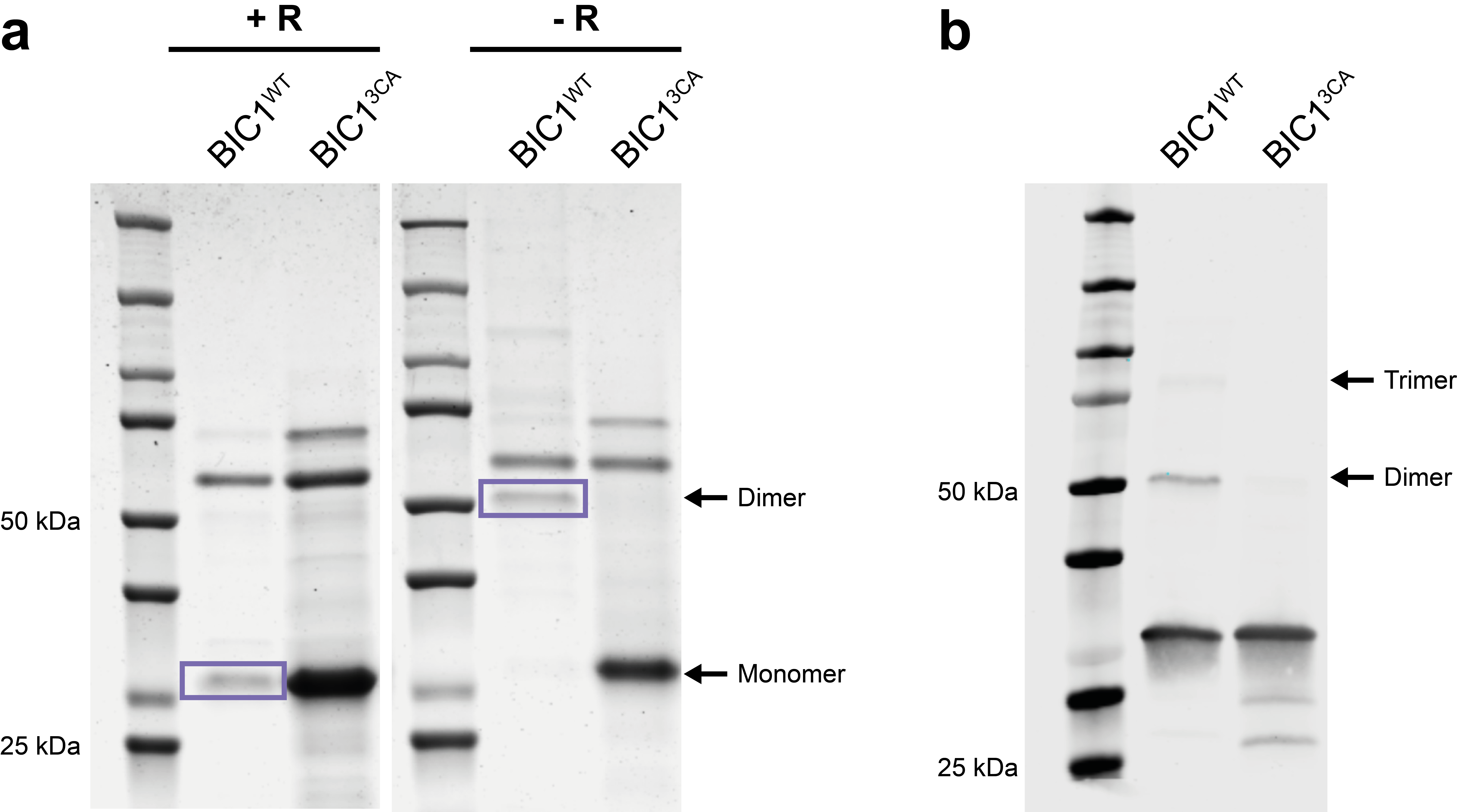


**Figure S8.** Wild-type BIC1 but not the BIC1^3CA^ triple mutant forms intermolecular disulfide bridges. a) Coomassie-stained SDS-PAGE gel with 3 µg of protein loaded in each lane of wild-type (BIC1^WT^) or the C to A triple mutant (BIC1^3CA^) in presence (+R) or absence (-R) of 10 mM reductant Tris(2-carboxyethyl)phosphine-hydrochloride – TCEP in SDS loading buffer, boiled for 10 min. The BIC1 monomers run at about 25 kDa. Black arrows indicate monomers, dimers or trimers of BIC1. b) Western blot of SDS-PAGE gel with 1.5 µg of protein loaded per lane and probed with αHis antibody showing that, in contrast to BIC^WT^, the BIC^3CA^ triple mutant does not form oligomers.
